# Comparing the Effects of Ginger and Glibenclamide on Dihydroxybenzoic Metabolites Produced in Stz-Induced Diabetic Rats

**DOI:** 10.5812/ijem.10266

**Published:** 2013-10-21

**Authors:** Ramesh Ahmadi, Saeede Pishghadam, Fatemeh Mollaamine, Mohammad Reza Zand Monfared

**Affiliations:** 1 Department of Physiology, Islamic Azad University, Qom, IR Iran; 2 Islamic Azad university, Qom, IR Iran; 3 Department of chemistry, Islamic Azad university, Qom, IR Iran

**Keywords:** Free Radical, STZ –Induced Diabetic Rats, Ginger, Glibenclamide

## Abstract

**Background:**

The aim of the present study was to investigate the effect of ginger and glibenclamide on oxidative stress markers. Oxidative stress is caused by an unbalance between a relative overload of oxidants and depletion of antioxidants, as implicated in the pathogenesis of several chronic diseases, including atherosclerosis and diabetes mellitus. Regarding the role of oxidative stress in the pathogenesis of diabetes mellitus, we investigated the effect of ginger and glibenclamide in diabetic rats induced bystreptozocin (STZ).

**Objectives:**

This study assessed the effects of ginger and glibenclamide on dihydroxybenzoic acid metabolites in diabetic rats.

**Materials and Methods:**

In this study 30 Wistar strain male rats were divided into five groups: Group 1: Normal control receiving normal saline (0.9 0/0), Group 2: control DMSO (Dimethyl sulfoxide) (as solvent of glibenclamide), Group 3: Diabetic control receiving Streptozocin (STZ ) (50 mg/kg) ,Group 4: diabetic+ Ginger Extract: this group received ginger ethanolic extract (200 mg/kg) via IP (Intraperitoneally) injection for 30 days, and Group 5 diabetic rats received glibenclamide (0.5 m/kg). Production of hydroxyl radicals was examined in the diabetic rats induced by streptozocin. Hydroxyl radicals were generated in plasma of the hyperglycemic rats, and were quantitatively assayed by trapping hydroxyl radicals with salicylic acid so as to produce 2,3-and 2,5-dihydroxybenzoic acid.

**Results:**

Production of hydroxyl radicals increased; therefore, by using salicylic acid, hydroxyl radicals were trapped and 2,3dihydroxybenzoic acid and 2,5dihydroxybenzoic acid metabolites were formed then measured by HPLC and spectrophotometer. Rats receiving ginger extract and glibenclamide showed decreased level of metabolites compared to the diabetic controls (P <0/001). This means that antioxidants act as scavenger of free radicals.

**Conclusions:**

Comparative effect of ginger and glibenclamide also showed that glibenclamide has antioxidant effect as a scavenger of free radical, but ginger is more capable of eliminating them.

## 1.Background

Oxidative stress is caused by an unbalance between a relative overload of oxidants and a depletion of antioxidants (1-4). In the research field of free radicals in biological samples it is still a major problem to determine the amount of free radical damage. One of the most aggressive radicals is the hydroxyl radical (5-7). Oxidative stress is currently suggested as the mechanism underlying diabetes and diabetic complications (8).

Hyperglycemia, several other factors like hyperlipidemia and enhanced oxidative stress play a major role in diabetic pathogenesis. The disease is progressive and is associated with high risk of complications (9). During diabetes, persistent hyperglycemia causes increased production of free radicals, especially reactive oxygen species (ROS), for all tissues from glucose auto-oxidation and protein glycosylation. Various mechanisms have been suggested to contribute to the formation of these reactive oxygen-free radicals. Glucose oxidation is believed to be the main source of free radicals (10). Pancreatic beta cells are particularly sensitive to be damaged via reactive oxygen species (ROS), whether generated by the complex pro inflammatory environment of an autoimmune cellular infiltrate (insulitis), or by the free radical generating toxin alloxan(11, 12). Oxidative stress is thought to be a major risk factor in the onset and progression of diabetes. Many of the common risk factors, such as obesity, increased age, and unhealthy eating habits, all contribute to an oxidative environment (13). Ginger (Zingiber officinale) is widely consumed as spice for the flavoring of foods. Ginger is reported to have several beneficial pharmacological effects (hypoglycemic, insulin tropic, and hypolipidemic) on health in humans (10) and in experimental animals (14, 15). It has been reported that ginger or its extracts possesses some pharmacological activities including anti emesis (16), analgesic effect (17), antitumor (18) and antioxidant (19). The active ingredients in ginger root include volatile oils and pungentphenol compounds known as gingerols, sesquiterpenoids, and shogaols (20). Zancan et al. mentioned that all major active ingredients of Z. officinale roots and leaves such as zingerone, gingerdiol, zingiberene, gingerols and shogaols have antioxidant activity (21). Since the effect of ginger and glibenclamide have not been compared for their antioxidant action on diabetic rats ,thus, the present study aimed to investigate comparative effect of ginger and glibenclamide on oxidative damage in streptozocin induced diabetic rats.

## 2.Objectives

The aim of the study was to compare the effects of ginger and glibenclamide on dihydroxybenzoic acid metabolites produced in diabetic rats.

## 3. Materials and Methods

### 3.1. Animals

Wistar male rats weighing 250±25 g were purchased from Karaj Pastor Institute. Rats were housed in clean plastic cages of six rats per each. Ambient temperature of animal room was (27 ± 2 °C) with a 12 hours dark and light cycle (22). Rats were given standard pellets diet and water (22) throughout the experimental period.

### 3.2. Chemicals

STZ was obtained from Sigma chemicals (The USA).

#### 3.2.1. Induction of Diabetes

The animals were fasted overnight and diabetes was induced by a single in traperitoneal injection of a freshly prepared solution of streptozocin (STZ) dissolved in physiological saline (50 mg/kg body weight).The animals were considered as diabetic, if their blood glucose values were above 250mg/dL on the third day after the STZ injection. The blood glucose was measured by glucometer.

### 3.3. Ginger Ethanolic Extract Preparation

Two kilograms of fresh rhizomes of ginger was purchased from a local market, peeled, washed, dried, and pulverized with a blender to a fine powder, and extracted in Soxhlet extractor with 95% ethanol for 24 hours,and the extraction was continued, filtered and the filtrate was concentrated to dryness under reduced pressure in arotary evaporator and then the extract was transported to Desiccator. The result in get hanolic extract was air-dried; finally resulting 80 grams of dark brown, gelatinous extract of gingerdried rhizomes. Without any further purification, the crude ethanolic extract was used for the experiments. Dose equivalent to 200 mg of the crude extract per kg body weight, was calculated(22).

### 3.4. Grouping of Animals

The rats were divided into five groups, six rats in each group and treated as follows: Including, normal control group (NC) which received normal saline (0.9%), and fed with normal diet, normal control which received dose of 0/5 mg/kg of DMSO (Dimethyl sulfoxide) (as a solvent of glibenclamide) and diabetic control group which was given streptozocin (STZ) intra peritoneally with a single dose of 50 mg/kg, and diabetes plus dose of 200 mg/kg ginger extract (D+Gli) and diabetics rats which were treated with a dose of 1 mg/kg glibenclamide for 30 days.Salicylic acid (22 mg/kg body weight) was injected to each rat. One hour after salicylic acid injection, rats were killed and blood was drawn from the left ventricle of heart and collected in a heparin-containing tube. By centrifuging at 2500(rpm) for 20 min, plasma (1000λ) was prepared for the measurement of hydroxyl radicals. Plasma was mixed with distilled water (10 times). The supernatant was filtered through a 0/45, 0/22 (μm) filter and assayed by HPLC.

### 3.5. Determination of 2,3-and 2,5-DHBA by HPLC (23)

The filtered sample (0.45 and 0.22 μm) was applied on to an HPLC apparatus, composed of a V7603 HPLC pump from, and a V7604 UV detector from KNAVER equipped with a flow cell operating at 280 and 305 nm. The analyses were performed at room temperatures using aRP18 250×4.6 mm SS EXSIL ODS 5μm and a flow rate mobile phase (Me OH:10/01 aqueous HOAC (80:20) 1mL/min.

### 3.6. Statistical Analysis

Analysis of one way ANOVA was used. Data were analyzed using the SPSS (Version SPSS15). Statistical significance was set at P < 0.05.

## 4. Results

### 4.1. Effect of Ginger and Glibenclamide on the Blood Glucose Levels and Body Weight Changes

The STZ-induced diabetic rats showed significant increase of blood glucose levels in comparison to normal control rats, which further increased during the experimental period. Intra peritoneally injection of ginger showed drastic increased levels in blood glucose in diabetic group, and body weight was lower than the control group (Table 1). 

**Table 1.** Blood Glucose Levels and Body Weight Changes in STZ-induced Rats Followed by Ginger ****and Glibenclamide Treatment.

Group	Blood Glucose, nmol/mL^a^		Body Weight, g^a^	
	0 th Day	30 th Day	0 th Day	30 th Day
**Group 1 (NC)**	78 ± 2.2	98 ± 1.8	212 ± 8.66	239 ± 25
**Group 2 (DMSO)**	100 ± 1.1	105 ± 2.3	204 ± 20	245 ± 19.1
**Group 3 (DC)**	463 ± 2.2^b^	501 ± 2.14^b^	181 ± 15.94^b^	211 ± 10.5
**Group 4 (Gt)**	455 ± 3.25^c^	260 ± 2.2	211 ± 11.3	212 ± 47^c^
**Group 5 (Gilt)**	377 ± 3.3^c^	202 ± 2.2^c^	193 ± 19.7	216 ± 32^c^

^a^(P < 0.01)

^b^All the values are mean ± SD of five individual observations.

^c^Values are significant compared to the normal control (P < 0.001) and diabetic control

### 4.2. Comparing the Production of 2, 3DHBA in Value Between the Groups

**Figure 1.**
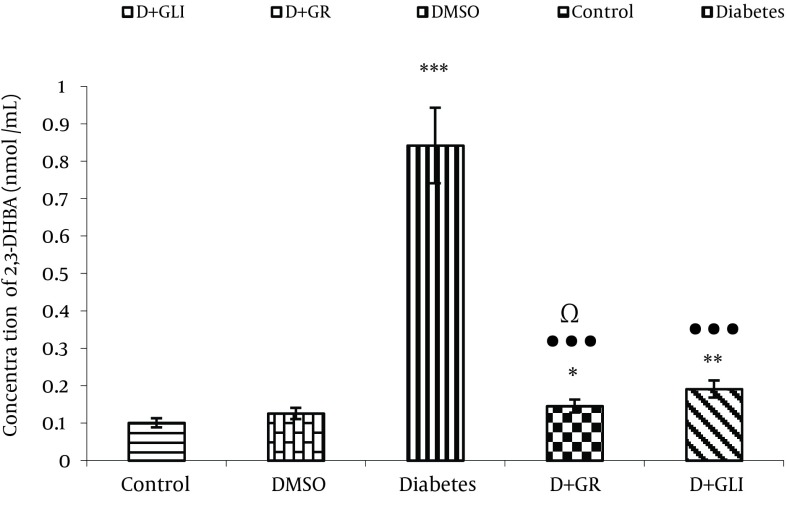
Comparing the Production of 2, 3 DHBA Between the Groups * shows the comparison between the control group and all other groups. ● shows the comparison between the treatment groups with diabetic control. Ω shows the comparison between the treatment groups.

Figure 1 Represents the Activity of Ginger Antioxidant and Decreased Level of2, 3-DHBA Metabolite in Rats Induced Diabetes Treated by Ginger Compared to glibenclamide (P < 0.01) and Diabetic Groups(P < 0.001). 

### 4.3. Comparing the Production of 2, 5DHBA Between the Groups

**Figure 2.**
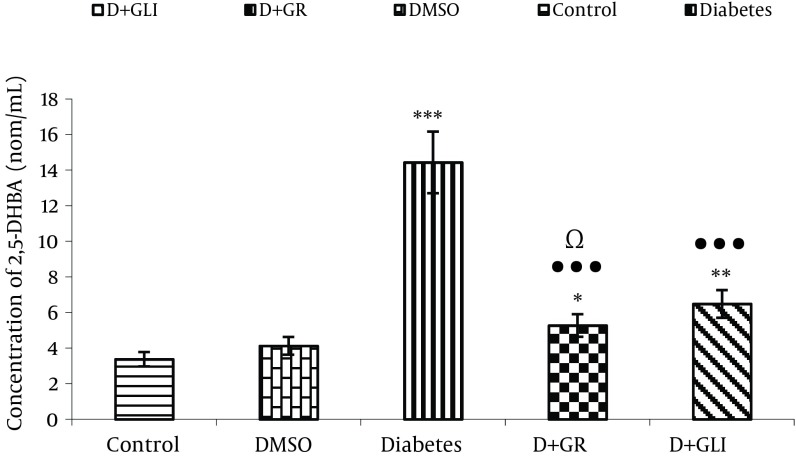
Comparing the Production of 2, 5 DHBA Between the Groups

Figure 2 also shows the activity of antioxidant of ginger in low production of 2, 5 DHBA compared to glibenclamide ( P < 0.01) and diabetic group (P< 0.001). 

## 5. Discussion

Our experimental findings showed that diabetic rats which received ginger extract had increased levels of 2, 3-DHBA and 2, 5-DHBA metabolites (P < 0.01). The antioxidant compounds and other pharmacological compounds of ginger may inhibit the production of free radicals. After 30 days, supplementation of ethanolic extract of ginger to diabetic rats, resulted in more significant diminution of 2,3-DHBA and 2,5-DHBA levels than diabetic rats administrated glibenclamide.

In STZ-induced diabetic rats hydroxyl radicals are increased significantly in plasma and it is detectable by increased levels of 2,3-DHBA and 2,5-DHBA. The present study clearly demonstrated that hydroxyl radical formation was markedly enhanced in plasma by induction of hyperglycemia with STZ. Diabetes is associated with a higher oxidative stress. ROS induced damage to the insulin producing pancreatic beta-cells induces diabetes (24). Salicylate has good properties to actas an in vivo marker for oxidative determine salicylate and its free radical products stress (25). Hydroxyl free radical may react with salicylate to form 2.3-dihydroxybenzoic acid(2.3DHBA) A and 2.5-dihydroxybenzoic acid (2.5DHBA) (23). Intermittent increases in ambient glucose concentrations are associated with increased production of reactive oxygenspecies (ROS) in vivo as well as in cell cultures(26-28). Diabetes mellitus affects approximately 100 million people (29), and its prevalence has been increasing (30). Oxidative stress can be defined as a state of imbalance toward the factors that generate reactive oxygen radicals (e.g., superoxide or hydroxyl radicals) (24). Under conditions of oxidative stress, free radicals that are not reduced or removed from the cellular environment can cause damage to all cellular macromolecules including nucleic acids, lipids, and proteins (31). Streptozocin injection resulted in diabetes mellitus, which may be due to the destruction of beta cells of Islets of Langerhans as proposed by others (32). Diabetes arises from irreversible destruction of pancreatic beta cells, causing degranulation and reduction of insulin secretion (33). During diabetes, persistent hyperglycemia causes increased production of free radicals, especially reactive oxygen species (ROS) (34). Glibenclamide eliminates reactive oxygen species (ROS)(35, 36) comparable with glibenclamide. Ginger is a standard hypoglycemic medication. Hydroxyl radical (OH) is the most reactive oxygen species (ROS), which can act with most organic molecules added to unsaturated bond, hydrogen abstraction, or electron transfer (its reduction potential 1.9 V) (37) Salicylate has good properties to act as an in vivo marker for oxidative stress (24).
