# Cooregendium: Comparing the Effects of Ginger and Glibenclamide on Dihydroxybenzoic Metabolites Produced in Stz-Induced Diabetic Rats [published Int J Endocrinol Metab. 2013;11(4):e10266]

**DOI:** 10.5812/ijem.19886

**Published:** 2014-04-01

**Authors:** Ramesh Ahmadi, Saeede Pishghadam, Fatemeh Mollaamine, Mohammad Reza Zand Monfared

**Affiliations:** 1 Department of Physiology, Qom Branch, Islamic Azad University, Qom, IR Iran; 2 Department Of Biology, Qom branch, Islamic Azad University, Qom, IR Iran; 3 Department of chemistry, Qom branch, Islamic Azad university, Qom, IR Iran

## corrigendum:

This corrigendum supplies corrected statements and proofs of an error by author. Hear by, authors declare that The correct authors list and affiliations are:

Ramesh Ahmadi ^1,*^, Saeede Pishghadam ^2^, Fatemeh Mollaamine ^3^ Mohammad Reza Zand Monfared ^3^

^1^ Department of Physiology, Qom Branch, Islamic Azad University, Qom, IR Iran

^2^ Department Of Biology, Qom branch, Islamic Azad University, Qom, IR Iran

^3^Department of chemistry, Qom branch, Islamic Azad university, Qom, IR Iran

